# Gastric cancer cell supernatant causes apoptosis and fibrosis in the peritoneal tissues and results in an environment favorable to peritoneal metastases, *in vitro* and *in vivo*

**DOI:** 10.1186/1471-230X-12-34

**Published:** 2012-04-20

**Authors:** Di Na, Zhi-Dong Lv, Fu-Nan Liu, Yan Xu, Cheng-Gang Jiang, Zhe Sun, Zhi-Feng Miao, Feng Li, Hui-Mian Xu

**Affiliations:** 1 Department of Oncology, The First Affiliated Hospital, China Medical University, Shenyang, 110001, Liaoning Province, China; 2 Department of Cell Biology, China Medical University, Shenyang, 110001, Liaoning Province, China

**Keywords:** Peritoneal carcinomatosis, Stomach neoplasms, Mesothelial cell, Apoptosis, Fibrosis

## Abstract

**Background:**

In this study, we examined effects of soluble factors released by gastric cancer cells on peritoneal mesothelial cells *in vitro* and *in vivo*.

**Methods:**

HMrSV5, a human peritoneal mesothelial cell line, was incubated with supernatants from gastric cancer cells. Morphological changes of HMrSV5 cells were observed. Apoptosis of HMrSV5 cells was observed under a transmission electron microscope and quantitatively determined by MTT assay and flow cytometry. Expressions of apoptosis-related proteins (caspase-3, caspase-8, Bax, bcl-2) were immunochemically evaluated.

**Results:**

Conspicuous morphological changes indicating apoptosis were observed in HMrSV5 cells 24 h after treatment with the supernatants of gastric cancer cells. *In vivo*, peritoneal tissues treated with gastric cancer cell supernatant were substantially thickened and contained extensive fibrosis.

**Conclusions:**

These findings demonstrate that supernatants of gastric cancer cells can induce apoptosis and fibrosis in HMrSV5 human peritoneal mesothelial cells through supernatants in the early peritoneal metastasis, in a time-dependent manner, and indicate that soluble factors in the peritoneal cavity affect the morphology and function of mesothelial cells so that the resulting environment can become favorable to peritoneal metastases.

## Background

Peritoneal carcinomatosis severely limits the improvement of gastric cancer patients’ prognoses after surgery [1]. Peritoneal metastasis results in a metastatic cascade, usually occurring at late-stage tumor development, which significantly contributes to gastric cancer-related mortality. The mechanisms of peritoneal metastasis of diffusely infiltrating carcinoma are not clearly understood.

The peritoneal stroma environment favors proliferation of tumor cells by serving as a rich source of growth factors and chemokines known to be involved in tumor metastasis. Molecules mediating this cascade have not been extensively investigated [2]. Reportedly, mesothelial cells could prevent cancer invasion and undergo morphological changes in response to soluble factors released by cancer cells [3]. The specific molecules involved in this process are dictated by intrinsic characteristics of the metastatic tumor cells. Tumor cells need to attach firmly onto the submesothelial monolayer and penetrate the mesothelial monolayer for invasion. Buck *et al.*[4] explored the protective effect of the mesothelial layer, using a rat model in which the mesothelial lining of parietal peritoneum was stripped off in the experimental rats, leaving the basal membrane intact. We previously showed that the layer of confluent, intact mesothelial cells hinders cancer cell invasion of the abdominal cavity. However, once the integrity of this barrier is disrupted, metastasis may occur, because the peritoneum provides a favorable environment for gastric cancer cells to grow [5-9]. Additional studies have shown that, prior to the attachment of gastric cancer cells onto peritoneum, mesothelial cells acquire hemispherical shape and start to shed. As a result, naked areas of the submesothelial connective tissue are exposed to the peritoneal cavity [10-12]. It is likely that penetration of the mesothelial monolayer by tumor cells initiates with tumor-induced mesothelial cell apoptosis. In this study, we examined the effects of soluble factors released by gastric cancer cells on morphology and biological activity of peritoneal mesothelial cells *in vitro and in vivo*.

## Methods

### Reagents

3-(4,5-Dimethylthiazol-2-yl)-2,5-diphenyl tetrazoliumbromide (MTT) was obtained from Fluka, USA. Propidium iodide (PI) was obtained from Biosharp, USA. Bcl-2, Bax, caspase-3, caspase-8 and actin primary antibodies, and the secondary antibody, goat anti-mouse IgG were obtained from Santa Cruz Biotechnology Inc., CA, USA. Dulbecco’s modified Eagle’s medium (DMEM) and fetal calf serum (FCS) were obtained from GIBCO BRL (Grand Island, NY, USA). Other laboratory reagents were obtained from Sigma, USA. A phase-contrast microscope (Japan Nikon), transmission electron microscope (Hitachi H-6001, Japan) was used.

### Cell lines and cell culture

The human peritoneal mesothelial cell line HMrSV5 was obtained from the Department of Cell Biology, China Medical University, China. HMrSV5 was originally isolated from human omentum. Briefly, omentum collected from consenting non-uremic patients who were undergoing elective abdominal surgery, and was incubated in 0.05 % (w/v) trypsin and 0.01 % (w/v) EDTA for 20 min at 37°C. The harvested mesothelial cells were centrifuged at 150 g for 5 min and then transferred into 75 cm^2^ tissue culture flasks and cultured in a humidified 5 % CO_2_ incubator, in DMEM supplemented with 10 % fetal calf serum (FCS), 100 U/mL penicillin, 100 μg/mL streptomycin, 2 mmol/L L-glutamine and 20 mmol/L hydroxyethyl piperazine ethanesulfonic acid (HEPES, GIBCo BRL, USA). Medium was changed every 2–3 days.

Human gastric carcinoma cell lines, MKN-45, MKN-1, SGC-7901, BGC-823 and MGC-803, were obtained from the Department of Cell Biology, China Medical University, China. These cells were cultured in DMEM supplemented with 10 % FCS, 100 U/mL penicillin, 100 μg/mL streptomycin, and 2 mmol/L L-glutamine in a humidified 5 % CO_2_ incubator at 37°C.

### Preparation of serum-free conditioned media

Serum-free conditioned media (SF-CM) was prepared from gastric cancer cells or normal gastric epithelial cells as previously reported [1]. Briefly, 5.0 × 10^5^ cells were seeded into 100-mm tissue culture dishes with 10-mL DMEM, supplemented with 10 % FCS and incubated at 37°C for 3 days. To obtain SF-CM, the cells were washed twice with phosphate-buffered solution (PBS) and then incubated for 2 days with 3 mL of serum-free DMEM. The SF-CM was eluted and centrifuged at 1000 g for 5 min, passed through filters (pore size: 0.45 μm) and stored at ^–^20°C until used.

### Animals

Male C57BL/6 mice (eight weeks old, weighing 20 ± 2 g), were obtained from China Medical University animal facility and fed with purified water and a commercial stock diet in an air-conditioned room at 20–22°C.Animals used in this study were maintained in accordance with the Guide for Care and Use of Laboratory Animals published by the US National Institutes of Health (NIH publication No. 85–23, revised 1996) and the Policy of Animal Care and Use Committee of China Medical University.

### Morphological Evaluation under a Phase-contrast Microscope

Human peritoneal mesothelial cells (HPMCs) were cultured to subconfluence in a 50-cm^2^ dish with DMEM containing 10 % FCS. The medium then was changed to (1) serum-free DMEM or (2) SF-CM from gastric cancer cell lines. The HPMCs in 24-well chambers were exposed to test solutions for 24 h, and gently washed with PBS. They were then examined under a phase-contrast microscope for size, shape, and integrity of the cell membrane, cytoplasm, and nucleus.

### Transmission electron microscopy

The HPMCs were cultured to subconfluence in a 50-cm^2^ dish with DMEM containing 10 % FCS. The medium then was changed to (1) serum-free DMEM or (2) SF-CM from gastric cancer cell lines. After incubation for 24 h, the cells were trypsinized and then fixed in ice-cold 2.5 % electron microscopy grade glutaraldehyde in PBS (pH 7.3). The specimens were rinsed with PBS, post-fixed in 1 % osmium tetroxide with 0.1 % potassium ferricyanide, dehydrated through a graded ethanol series (30 %–90 %), and embedded in Epon. Semi-thin (300 nm) sections were cut using a Reichart Ultracut (Reichart Ultracut (Leica, Germany), stained with 0.5 % toluidine blue, and examined under a light microscope. Ultrathin sections (65 nm) were stained with 2 % uranyl acetate and Reynold’s lead citrate, and examined on a transmission electron microscope at × 5000 or × 8000 magnification.

### Quantitative determination of cell damage by MTT assay

The MTT assay was performed to assess viability of the human peritoneal mesothelial cells after treatment with SF-CM from gastric cancer cell lines. Cells in 96-well plate cultures, after exposure to control or test solutions for a specific time period (observed at 12 h, 24 h and 48 h), were incubated with 50 μg/mL MTT at a dilution of 1:10, based on the volume of culture medium for 3 h at 37°C. At the end of the incubation time, the MTT solution was removed and 150 μL DMSO was added to each well, and stirred to dissolve the dark-blue formazon crystals which had formed. The proportion of viable cells was determined by measuring the optical density of each sample at 480 nm with a spectrophotometer. Three cultures were exposed to each solution at each time period. The means of each group of cultures were compared.

### Flow cytometry

Following incubation in the test solutions for 24 h, 48 h and 72 h, cells were harvested using trypsinization. Cells were resuspended in PBS at a concentration of 1 × 10^6^/mL and fixed in 2 mL methanol for 30 min at 4°C. After the CNE2 cells were fixed, the mixture was incubated in 0.5 mL of PI solution (0.05 mg/mL in 3.8 mol/L Na citrate) and 0.5 mL of RNAse A (0.5 mg/mL) at room temperature for 30 min. Finally, the cells were resuspended in 1 mL PBS and analyzed by flow cytometry according to the manufacturer’s instructions. The cells in the subdiploid peak were considered apoptotic.

### Histological appearance of peritoneum

Male C57BL/6 mice (eight weeks old, weighing 20 ± 2 g) were used in the present study. The experiment followed the guidelines for the use of Laboratory Animals in Research and Teaching. The mice were fed a standard pellet laboratory chow and were provided with water *ad libitum*. Mice were randomly assigned to one of three groups (n = 5 or 6 in each group). Mice in the DMEM group were treated with DMEM (100 ml/Kg) by intraperitoneal injections on days 1, 3, 5 and 7. Mice in the SF-CM group were treated with 100 ml/Kg of SF-CM from gastric cancer cell lines by intraperitoneal injections on days 1, 3, 5 and 7. Nothing was added to injections for the control mice. After 29 days, mice were anesthetized with ethyl ether and sacrificed. Parietal peritoneums were stained with hematoxylin and eosin (H&E) and Masson’s trichrome staining. Morphologic changes of the parietal peritoneum were observed by light microscope. Thickness of the submesothelial extracellular matrix was determined after the tissue sections had H&E and Masson staining. The average for 10 independent measurements was calculated for each section; data were then summarized.

### Western blotting

Human peritoneal mesothelial cells were cultured to subconfluence in a 50-cm^2^ dish with DMEM containing 10 % FCS. The media were then changed to (1) SF-CM from gastric cancer cell MKN-45, MKN-1, SGC-7901, BGC-823 and MGC-803; and (2) serum-free DMEM serving as control. Protein was extracted in a standard lysis buffer with proteinase inhibitors (sodium orthovanadate, phenylmethylsulfonyl fluoride, leupeptin, and aprotinin obtained from BioShop, Burlington, ON, Canada). Aliquots of 20 μg of protein lysate was electrophoresed with a 12 % SDS-PAGE gel, transferred to a nylon membrane, and separately probed with antibodies for Bcl-2, Bax, caspase-3 and caspase-8. Following incubation with the secondary antibody, blots were developed using an ECL Western Blot Substrate Kit (Abcam, USA).

### Statistical analysis

All data are expressed as *x* ± *sd*. Comparisons of drug effects were made using Student’s *t*-test. A *P* value < 0.05 was considered to be significant.

## Results

### Morphological evaluation under a phase-contrast microscope

**Figure 1:**
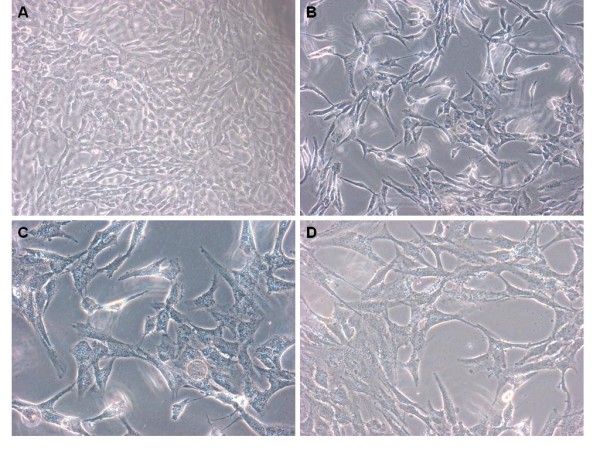
Morphological changes of human peritoneal mesothelial cells under phase-contrast microscope. **(A)** Monolayer of polygonal and cobblestone-like HPMCs cultured in serum-free DMEM. **(B–D)** Exfoliated appearance of naked areas after treatment with SF-CM from gastric cancer cell lines MKN45, SGC7901, and BGC823. (Magnification: ×40).

While HPMCs treated only in serum-free DMEM showed a typical polygonal and cobblestone pattern (Figure 1A.), cells treated with SF-CM from gastric cancer cell MKN45 for 24 h began to have morphological changes, the most obvious of which were exfoliation and the appearance of naked areas (Figure 1B).

### Transmission electron microscopy

**Figure 2:**
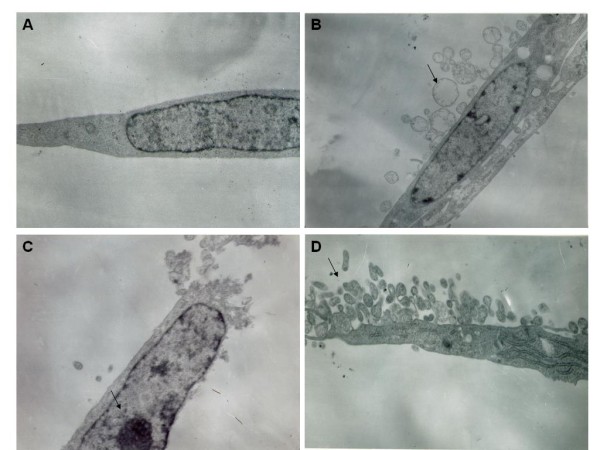
Human peritoneal mesothelial cells (HPMC) 24 h after incubation with and without SF-CM from gastric cancer cells. **(A)** Control cells display normal nuclei and endoplasmic reticula. **(B)** Cells treated with SF-CM from MKN1 gastric cancer cells show chromatin condensed into masses, and on the boundary of the membrane or forming arch. Budding and the formation of the apoptosis bodies were observed (arrows in **B**). **(C)** .Cells treated with SF-CM from MKN45 gastric cancer cells show condensation of nuclear chromatin (arrows in **C**). **(D)** Cells treated with gastric cancer cell line MGC-803 were similar to B. Budding and the formation of the apoptosis bodies were also observed.

After 24 h of SF-CM from gastric cancer cell treatment, apoptotic features (such as condensation of the nuclear chromatin, wrinkling of nuclear membranes, dilation of endoplasmic reticulum, and relatively normal structure of the mitochondria) were observed under transmission electron microscope (TEM; Figures 2A, B). Under TEM, the nuclear membrane was seen to be intact, the chromatin condensed into masses, and on the boundary of the membrane or forming arch (Figure 2C). The budding and the formation of the apoptosis bodies were also observed (Figure 2C).

### MTT assay

**Figure 3:**
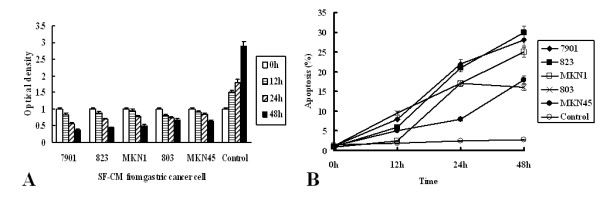
Apoptosis was quantified by two methods: MTT and flow cytometry. **(A)** Viability of mesothelial cell HMrSV5 after treatment with SF-CM from gastric cancer cells. **(B)** Apoptosis was quantified by flow cytometry after treatment with SF-CM from gastric cancer cells.

To evaluate potential suppressive effects of gastric cancer cell SF-CM on HPMCs, we examined its growth curve on the HPMC line HMrSV5. Gastric cancer cell SF-CM induced growth suppression in HPMC cells, and did so in a time-dependent manner (Figure 3A). This effect was observed at 0 h, 12 h, 24 h and 48 h. These results indicate that tumor supernatant induces mesothelial cell damage or apoptosis.

### Flow cytometry

To quantify the percentage of apoptotic cells after treatment at various time periods, mesothelial cells were stained with PI. Gastric cancer cell SF-CM effectively induced apoptosis in mesothelial cells and did so in a dose-dependent manner after 48 h (Figure 3.B). These results were the same as those for the MTT assay.

### Histology and morphometric analysis

**Figure 4:**
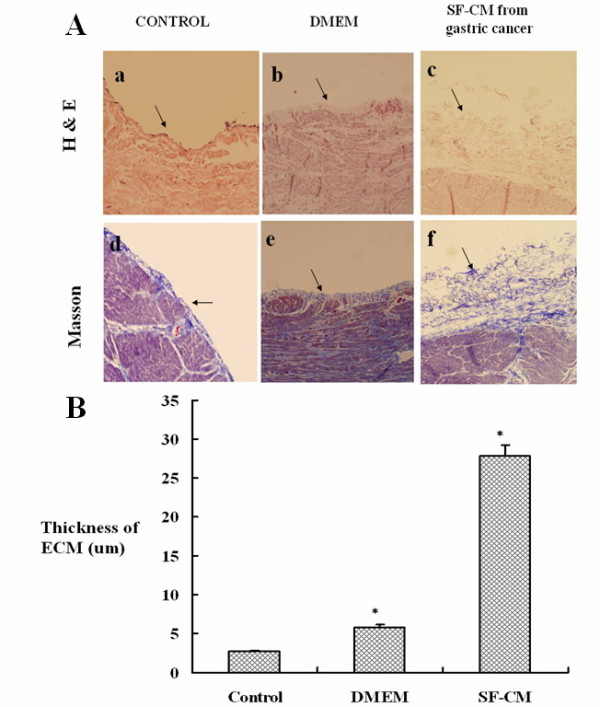
Hematoxylin/eosin (H&E) and Masson staining of peritoneum tissues. Peritoneum tissues from different groups were obtained surgically and subjected to H&E and Masson staining. **(A)** All photos were obtained at 40 × magnification. Normal peritoneum consists of only a monolayer of mesothelium with little fibrosis (a, d). Peritoneum treated with DMEM also showed small amounts of connective tissue under the mesothelial cells(arrows in b,e). In contrast, peritoneum treated by SF-CM from gastric cancer cells was substantially thickened and contained extensive fibrosis (arrows in c,f). **(B)** Morphometric parameters of peritoneal tissues. Data are expressed as the mean ± standard error of the mean of at least 3 separate experiments. *P < 0.05.

Morphologic changes of the parietal peritoneum were analyzed using H&E and Masson’s trichrome staining. Among normal mice, a mesothelial cell monolayer covered the peritoneal surface without any thickening (Figure 4 a,d). Due to apparent incompatibility, mice receiving intraperitoneal injections of DMEM had slight thickening in the peritoneal submesothelial collagenous zone (Figure 4 b, e); those injected intraperitoneally with gastric cancer cell SF-CM had marked thickening of the submesothelial compact zone and increased cellularity (Figure 4 c, f).

### Western blotting

**Figure 5:**
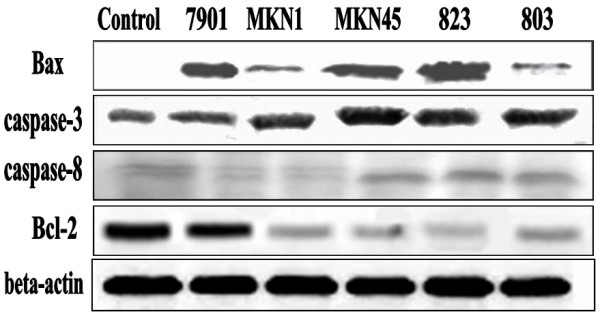
Western blot analysis of apoptosis-related protein levels (caspase-3, caspase-8, Bax, and bcl-2) in HPMCs with SF-CM from different gastric cancer cell lines treatment. Serum-starved HPMCs were incubated with SF-CM from different gastric cancer cell lines for up to 48 h; total cellular protein was extracted and subjected to western blot analysis.

We then sought to further delineate the mechanisms which underlie the combined effects of gastric cancer cell SF-CM on apoptosis-related proteins (caspase-3, caspase-8, Bax, bcl-2). Levels of these proteins were evaluated using western blot analysis. Caspase-3, caspase-8, and Bax protein levels increased after 48 h of treatment with SF-CM from most gastric cancer cells, while bcl-2 protein levels decreased (Figure 5). Beta-actin was used as the loading control.

## Discussion

Most studies of post-operative tumor recurrence show that traumatized mesothelial surfaces are preferred sites for tumor cell adhesion. Recently, disassociated cancer cells inside peritoneal cavities, and proteins specifically expressed in peritoneal metastasis of gastric carcinoma were found to be linked to cancer prognoses. While immunogenetic approaches show great promise in the treatment of peritoneal metastasis of gastric carcinoma [13-15], the effects of gastric cancer cells on mesothelial cells are poorly understood.

Study showed that mesothelial cells provided protection against peritoneal metastasis of tumor in intact mesothelia [9,16,17]. Paget *et al* proposed a “seed and soil” theory: metastasis only occurs when tumor cells live and grow in a favorable environment [18]. The peritoneum might be such a favorable environment for scirrhous gastric cancer cells; possibly mesothelial cells prevent cancer cells from infiltrating into submesothelial connective tissue. Masakazu *et al.*[1] showed that adjacent confluent mesothelial cells hindered invasion by cancer cells. In addition, Kiyasu *et al.*[3] reported that, prior to peritoneal implantation of cancer cells, mesothelial cells become hemispherical and exfoliate from the peritoneum. Our hypothesis is that after serosa are exposed, free cancer cells shed from primary gastric cancer sites into the abdominal cavity induce apoptosis in peritoneal mesothelial cells [19-21]. As a result, mesothelial cells become hemispherical and exfoliation takes place. Naked areas of submesothelial connective tissue are thus exposed to the peritoneal cavity; this injured peritoneal site becomes a favorable environment for peritoneal metastasis [22-24].

We had previously shown gastric cancer cell supernatant to significantly reduce the viability of mesothelial cells, but normal gastric epithelial cell line GES-1 exerts no effect on mesothelial cells [5]. Our present study also demonstrates that cultured mesothelial cells become hemispherical, and exfoliation occur when serum-free medium conditioned by gastric cancer cells was added, as shown by contrast phase microscopy. Furthermore, cytoplasmic reduction, nuclear condensation, and formation of extracellular and/or intracellular apoptotic bodies were observed under transmission electron microscope. Apoptosis was quantified by two methods: MTT and flow cytometry. We speculate that free gastric cancer cells in the abdominal cavity induce apoptosis of mesothelial cells and cause exfoliation, eventually leading to metastasis. This may be the mechanism by which cancer cells adhere to submesothelial connective tissue, although the mesothelial cells are still well-organized and confluent. Further studies are needed to characterize SF-CM released from gastric cancer cells.

Gastric cancer cells may induce apoptosis through mitochondria- and death receptor-dependent apoptotic pathways. Gastric cancer cells suppress mesothelial cell growth by inhibiting proliferation through the promotion of caspase-dependent apoptosis. Caspases are cytoplasmic aspartate-specific cysteine proteases, and play important roles in apoptosis [25]. The death receptor-dependent apoptotic pathway is triggered at the cell surface and requires activation of caspase-8, whereas the mitochondrion-dependent pathway is initiated by the release of mitochondrial cytochrome c into the cytoplasm and requires activation of caspase-9. Subsequently, caspase-8 or −9 can activate caspase-3, which in turn targets and degrades specific and vital cellular proteins, ultimately resulting in nuclear DNA degradation and apoptotic cell death [26]. Bcl-2, an inhibitor of the mitochondrial apoptosis pathway, exerts its action by blocking proapoptotic counterparts, which in turn prevents the release of cytochrome c and the activation of caspases [27]. Bax is a death promoter, which is neutralized by heterodimerization with Bcl-2. Bax translocates into the outer mitochondrial membrane followed by leakage of cytochrome c from the mitochondria into the cytosol [28]. Caspase-9 and caspase-3 are activated sequentially, and these events lead to the breakdown of chromosomal DNA. As there is a significant possibility that gastric cancer cell-mediated apoptosis of mesothelial cells is the result of regulation of Bcl-2 and Bax, identification of their target compounds is necessary.

In this study, we utilized a mouse experimental model of peritoneal sclerosis induced by repeated injections of gastric cancer cell SF-CM. Experimental peritoneal fibrosis induced by repeated intraperitoneal injections of gastric cancer cell SF-CM might not completely mimic peritoneal sclerosis observed in patients (diffusely infiltrating carcinoma or Bormann’s Type VI carcinoma). In fact, pathologic findings of peritoneal carcinomatosis and peritoneal sclerosis are not uniform and various factors are involved. In addition, certain common features are observed during the development of peritoneal sclerosis between gastric cancer cell SF-CM-induced experimental animal models and human patients undergoing peritoneal carcinomatosis. These common histological findings include increased accumulation of interstitial collagens such as type I and III collagen, infiltration of monocytes/macrophages, increase in a-SMA^+^ myofibroblasts, and vascular density in the peritoneum [7,8]. These similarities in alterations of the peritoneal membranes between experimental models and human peritoneal carcinomatosis patients suggest that this is an appropriate model for examining the efficacy of various potential therapeutic reagents for regulating peritoneal carcinomatosis.

## Conclusions

These findings demonstrate that gastric cancer cells can induce apoptosis and fibrosis of human peritoneal mesothelial cells through supernatants in the early peritoneal metastasis, and indicate that soluble factors in the peritoneal cavity can affect the morphology and function of mesothelial cells so that the resulting environment becomes favorable to peritoneal metastases.

## Abbreviations

HPMCs, Human peritoneal mesothelial cells; SF-CM, Serum-free conditional media.

## Competing interests

The authors declare that they have no competing interests.

## Authors’ contributions

DN, Z-DL and F-NL carried out the studies on morphology and biological activity of peritoneal mesothelial cells *in vitro* and *in vivo*. ZS, Z-FM and FL participated in the *in vivo* studies. YX and C-GJ participated in the morphology studies. DN and HX participated in the design of the study and performed the statistical analysis. HX and DN conceived of the study, and participated in its design and coordination and helped to draft the manuscript. All authors read and approved the final manuscript.

## Pre-publication history

The pre-publication history for this paper can be accessed here:

http://www.biomedcentral.com/1471-230X/12/34/prepub
